# Sleep health practices and sleep knowledge among healthcare professionals in Dutch paediatric rehabilitation

**DOI:** 10.1111/cch.12799

**Published:** 2020-08-12

**Authors:** Raquel Y. Hulst, Sigrid Pillen, Jeanine M. Voorman, Neele Rave, Johanna M.A. Visser‐Meily, Olaf Verschuren

**Affiliations:** ^1^ Center of Excellence for Rehabilitation Medicine, UMC Utrecht Brain Center University Medical Center Utrecht, Utrecht University and De Hoogstraat Rehabilitation Utrecht The Netherlands; ^2^ Sleep Medicine Center Kempenhaeghe Heeze The Netherlands; ^3^ Department of Electrical Engineering Technical University Eindhoven Eindhoven The Netherlands; ^4^ Department of Rehabilitation, Physical Therapy Science and Sports, UMC Utrecht Brain Center University Medical Center Utrecht Utrecht The Netherlands

**Keywords:** healthcare providers, neurodevelopmental disorders, paediatric rehabilitation, sleep education, sleep health practices, sleep knowledge

## Abstract

**Background:**

Sleep disorders are highly prevalent in children with neurodisabilities, yet they seem under‐recognized in paediatric rehabilitation settings. The aim of this study was to assess among two groups of healthcare professionals (HCPs) working in paediatric rehabilitation: (1) sleep health practices and (2) knowledge about sleep physiology, sleep disorders and sleep hygiene.

**Methods:**

We performed a cross‐sectional sleep survey among medical and non‐medical HCPs and the general population. Participants (30 rehabilitation physicians [RPs], 54 allied health professionals [AHPs] and 63 controls) received an anonymous 30‐item survey consisting of three domains: (1) general information, (2) application of sleep health practices and (3) sleep knowledge.

**Results:**

RPs address sleep issues more frequently in clinical practice than AHPs. Sleep interventions mostly consist of giving advice about healthy sleep practices and are given by the majority of HCPs. While RPs demonstrated the highest scores on all knowledge domains, total sleep knowledge scores did not exceed 50% correct across groups, with AHPs and controls showing equal scores. Sleep hygiene rules closest to bedtime and related to the sleep environment were best known, whereas those related to daytime practices were rarely mentioned across all groups. A small minority of HCPs (RPs 20%; AHPs 15%) believed to possess sufficient sleep knowledge to address sleep in clinical practice. No association was found between self‐perceived knowledge and sleep knowledge scores among HCPs.

**Conclusions:**

Sleep should become a standard item for review during routine health assessments in paediatric rehabilitation settings. HCPs' limited exposure to sleep education may result in feelings of incompetence and inadequate sleep knowledge levels, affecting their sleep health practices. Appropriate sleep training programs should be implemented to empower HCPs with knowledge, skills and confidence, needed to recognize and treat sleep disorders in children with neurodisabilities, as well as to be able to guide parents.

**Key messages**

- Sleep problems are highly prevalent among children with neurodevelopmental disabilities (NDDs), yet they are under‐reported, under‐recognized, under‐diagnosed and often untreated.
- Limited exposure to sleep education likely contributes to low levels of sleep knowledge, and confidence to guide patients, among healthcare professionals.
- There is a need to educate and empower healthcare professionals with knowledge and skills that are required to address sleep problems in children with NDDs and their families.
- Sleep should become a standard item for review during routine health assessments in paediatric rehabilitation settings.

## 1 INTRODUCTION

Sleep disorders are common in children with neurodevelopmental disabilities (NDDs), with a reported prevalence as high as 86% (Robinson‐Shelton & Malow, 2016; Simard‐Tremblay, Constantin, Gruber, Brouillette, & Shevell, 2011). In addition to affecting the children's physical and cognitive health and development (Turnbull, Reid, & Morton, 2013), sleep disorders may greatly impact on the well‐being of both the children and their families (Adiga, Gupta, Khanna, Taly, & Thennarasu, 2014; Mörelius & Hemmingsson, 2014; Sandella, O'Brien, Shank, & Warschausky, 2011). Therefore, improving the quality of sleep in children with NDDs not only has the potential benefit of improving their clinical outcomes (Owens 2008), it can greatly ameliorate the quality of life of the entire family (McDonald & Joseph, 2019).

In recent years, there has been growing acknowledgement of the importance of sleep and the need for recognition of sleep problems by physicians (Perry et al., 2013). Yet there continues to be only limited education in sleep medicine across medical school curricula (McDonald & Joseph, 2019; Mindell et al., 2011). The minimal training received is accompanied by shortcomings in confidence and sleep knowledge, all of which may contribute to sleep issues not being inquired about routinely when children are seen (Chervin, Archbold, Panahi, & Pituch, 2001; Honaker & Meltzer, 2016).

For children receiving developmental or rehabilitative services, like those with NDDs, paediatric rehabilitation provides an ideal place to address their sleep health as part of the routine assessments. The multidisciplinary team of healthcare professionals (HCPs) that is typically involved in the rehabilitative care of these children thereby serves a joint role in both detecting and managing sleep problems. Hence, it is crucial that (non‐)medical HCPs working in paediatric rehabilitation settings are not only aware of the importance of sleep but also possess current knowledge of basic sleep physiology, can recognize symptoms of common paediatric sleep disorders, and are familiar with good sleep hygiene practices.

However, despite the high prevalence of sleep disorders in children with NDDs, according to parents, sleep has received only limited attention in paediatric rehabilitation (Hulst et al., 2020). Indeed, sleep problems are not always appropriately addressed in these populations (Didden, Korzilius, van Aperlo, van Overloop, & de Vries, 2002; Simard‐Tremblay et al., 2011), leaving sleep an underemphasised aspect of health in neurorehabilitation (Verschuren, Gorter, & Pritchard‐Wiart, 2017). This raises the question whether HCPs have sufficient knowledge and competence to address sleep issues in clinical practice. Therefore, this survey study aimed to assess the (1) sleep health practices and (2) sleep knowledge (sleep physiology, sleep disorders, and sleep hygiene) in two groups of HCPs (i.e., medical and non‐medical professionals) working within paediatric rehabilitation settings. To effectively guide parents, HCPs are required to have more sleep knowledge than the general population, and therefore, a control group was added to allow comparisons.

## 2 METHODS

### 2.1 Study design

A cross‐sectional quantitative survey study was conducted. The study was deemed exempt from review under the Dutch Medical Research Involving Human Subjects Act by the Medical Ethics Research Committee of the University Medical Centre Utrecht, the Netherlands (file number 19‐066).

### 2.2 Respondents

HCPs from three paediatric rehabilitation settings (rehabilitation centre, school for special education and rehabilitation department of a children's hospital) in the Netherlands participated in this study. In Dutch rehabilitation, a medically schooled physician serves a gatekeeping role in detecting child‐related problems during clinical encounters and consequently coordinates the child's rehabilitative care. When, in this case, a problem with sleep of the child is detected, the physician gives first‐line treatment advice or can decide to set up referral to a non‐medical professional or sleep clinic for subsequent sleep assessments and/or interventions. Depending on the nature of the sleep problem, a non‐medical professional may further assess the child's sleep, bed routine and/or behaviour and perform therapies like behavioural interventions and implementing healthy sleep practices. In this way, the roles of HCPs involved in sleep care in Dutch rehabilitation settings are distinct. The pen‐and‐paper surveys were distributed during live meetings among the following two groups of paediatric HCPs:

1. Rehabilitation physicians (RPs). This group included paediatric rehabilitation physicians, physician assistants, paediatricians and doctors in specialist training to become RP.
2. Allied health professionals (AHPs). This group included physical therapists, occupational therapists, developmental behavioural therapists, speech and language therapists, social workers and psychologists.

An additional control group was drawn from the general population, comprising individuals without a background or current profession in healthcare, to allow comparisons of sleep knowledge levels. The control group consisted mostly of parents of (young) children, who were recruited via the social networks of colleagues and acquaintances, and filled out the pen‐and‐paper surveys during face‐to‐face encounters.

### 2.3 Data collection

A 30‐item structured questionnaire was designed based on relevant literature and consultations with experts (researchers and clinicians) working in the field of paediatric rehabilitation and sleep medicine (Supporting Information). Pilot testing was conducted to ensure that the questionnaire was easily understood and could be completed within a short time window (15–20 min). The questionnaire comprised three sections:

1. general information including age, sex, profession, educational level and hours of sleep education received;
2. application of sleep health practices in daily clinical practice. HCPs were asked to indicate how often they address the topic sleep during clinical encounters, choosing between *never/seldom* (less than once per month), *sometimes* (1–3 times per month) or *often* (once per week or more often). HCPs who reported to address sleep *sometimes* or *often* in clinical practice were asked to indicate the type(s) of sleep interventions or therapies they apply.
3. sleep knowledge within the domains of (a) basic sleep physiology (i.e., recommended sleep durations and sleep architecture), (b) symptoms and characteristics of common paediatric sleep disorders and (c) sleep hygiene rules (i.e., healthy sleep practices). Additionally, the participants' self‐perceived knowledge sufficiency was assessed.

Apart from the general information, responses were measured using multiple choice questions and included a ‘*don* *'* *t know*’ answer option. An open‐ended question was used for collecting information about knowledge of sleep hygiene rules; respondents were asked to name three sleep hygiene rules other than the example given regarding limiting screen time 2 h before bedtime. A cover letter that explained the aim of the study and emphasized the need for honest responses (i.e., to answer with ‘*don't know*’ instead of guessing if one does not know) was attached to the survey.

### 2.4 Data analysis

Surveys that returned largely incomplete (>25%) were excluded from analysis. Data were analysed using IBM SPSS Statistics 26. Sleep knowledge scores (i.e., number of correctly answered questions) were calculated for each group and converted into percentages; these are presented as mean ± standard deviation (SD)% scores for all knowledge questions in total and per domain sleep physiology and sleep disorders. After testing for normality, means were compared using Kruskal–Wallis test followed by post hoc Mann–Whitney tests. Answers to the open‐ended question regarding sleep hygiene rules were categorized according to those presented by the National Sleep Foundation (2019), and relative frequencies were calculated per group. Categorical variables (i.e., sleep education, sleep health practices and knowledge about sleep hygiene rules) are displayed as percentage frequency distributions. To determine the relationship between categorical data, relative frequencies were compared using Fisher's exact tests. The critical value for significance was set at 0.05, and a correction for multiple comparisons was applied during post hoc analyses.

## 3 RESULTS

### 3.1 Respondents' general information

In total, 84 HCPs completed the survey. Based on their profession, HCPs were divided between the RP group (*n* = 30) and the AHP group (*n* = 54). An additional control group (*n* = 63) completed the survey questions with exception of the section regarding sleep health practices. The majority of all respondents were female and between the age of 31–50 years. Across all groups, over 75% indicated to have received less than 5 h of sleep education throughout their entire school curricula (RPs 75.9%; AHPs 90.6%; controls 88.5%), and this amount was independent of group (*p* = 0.102, Fisher's exact test). Group characteristics are summarized in Table 1.

**TABLE 1:** Group characteristics

	Rehabilitation physicians^a^ *n* = 30	Allied health professionals^b^ *n* = 54	Control group *n* = 63
	% (*n*)	% (*n*)	% (*n*)
Sex			
Female	76.7 (23)	92.6 (50)	73.0 (46)
Age			
20–30 years	33.3 (10)	13.0 (7)	31.8 (20)
31–40 years	40.0 (12)	33.3 (18)	23.8 (15)
41–50 years	16.7 (5)	24.1 (13)	27.0 (17)
51–60 years	10.0 (3)	24.1 (13)	14.3 (9)
61–70 years	0 (0)	5.6 (3)	3.2 (2)
Level of education			
Secondary vocational	0 (0)	0 (0)	15.9 (10)
Higher professional	3.3 (1)	77.8 (42)	20.6 (13)
University or higher	97.7 (29)	22.2 (12)	63.5 (40)

^a^ This group consisted of paediatric rehabilitation physicians (*n* = 9), doctors in specialist training to become RP (*n* = 19), physician assistant (*n* = 1), and paediatrician (*n* = 1).

^b^ This group consisted of physical therapists (*n* = 17), occupational therapists (*n* = 15), developmental behavioural therapists (*n* = 8), speech and language therapists (*n* = 7), social workers (*n* = 4) and psychologists (*n* = 3).

### 3.2 Sleep health practices

RPs (*often* 56.7%, *n* = 17; *sometimes* 40%, *n* = 12; *never/seldom* 3.3%, n = 1) reported to more frequently address sleep issues than AHPs (*often* 11.8%, *n* = 6; *sometimes* 51%, *n* = 26; *never/seldom* 37.2%, *n* = 19), a difference found to be statistically significant (*p* < 0.001*, Fisher's exact test).

### 3.3 Sleep interventions

**FIGURE 1:**
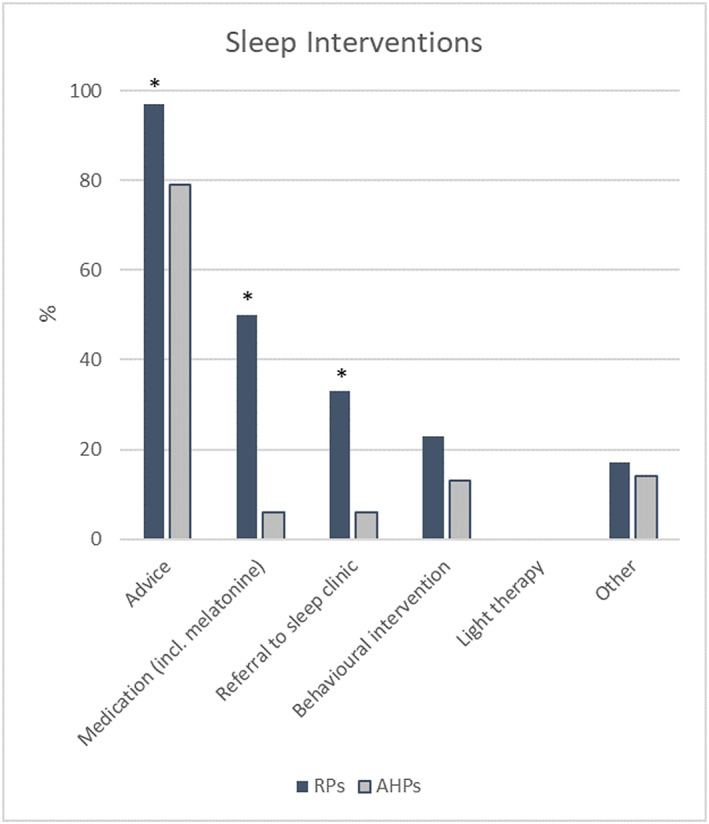
Types of sleep interventions applied by RPs (*n* = 30) and AHPs (*n* = 54). ^*^ *p* < 0.05 (Fisher's exact test); AHPs, allied health professionals; RPs, rehabilitation physicians [Colour figure can be viewed at wileyonlinelibrary.com]

Those who reported to *sometimes* or *often* address sleep indicated the types of sleep interventions they apply (Figure 1). The majority of both HCPs groups (RPs 97%, *n* = 29; AHPs 79%, *n* = 38) reported to give advice about sleep hygiene rules. RPs more often mentioned giving advice compared with AHPs (*p* = 0.043*, Fisher's exact test). Half of RPs (50%, *n* = 15) indicated to prescribe medication (including melatonin) compared with 6% (*n* = 3) of AHPs (*p* < 0.001*, Fisher's exact test). One third of RPs (33%, *n* = 10) indicated to refer to a sleep clinic, compared with 6% (*n* = 3) of AHPs (*p* = 0.004*, Fisher's exact test). Less than a quarter of HCPs (RPs 23%, *n* = 7; AHPs 13%, *n* = 6) reported to perform behavioural therapy to treat sleep problems (*p* = 0.229, Fisher's exact test). None of the HCPs reported to perform light therapy.

### 3.4 Sleep knowledge

#### 3.4.1 Sleep physiology and sleep disorders

Table 2 shows the sleep knowledge scores (correct answer rates) across groups. Mean total sleep knowledge scores were found to be statistically different across groups (*H*(2) = 24.322, *p* < 0.001*), with RPs demonstrating significantly higher scores than AHPs (*p* < 0.001*) and controls (*p* < 0.001*), whereas no difference was found between AHPs and controls (*p* = 0.667).

**TABLE 2:** Sleep knowledge and self‐perceived sleep knowledge scores

	Rehabilitation physicians *n* = 30	Allied health professionals *n* = 54	Control group *n* = 63
	mean ± SD	mean ± SD	mean ± SD
Sleep knowledge scores (%)			
Total sleep knowledge	48.9 ± 12.4^*^	35.4 ± 10.9	34.5 ± 13.3
Domain sleep physiology	53.3 ± 12.6^*^	37.4 ± 15.9	43.0 ± 16.3
Domain sleep disorders	41.3 ± 17.1^*^	26.6 ± 15.9^#^	19.4 ± 17.8
Self‐perceived sleep knowledge scores (%)			
Total sleep knowledge score in response to the question: ‘Do you believe you have sufficient sleep knowledge to address sleep issues in clinical practice?’			
Yes	54.76 ± 9.87 (*n* = 6)	34.52 ± 7.94 (*n* = 8)	N/A
No	50.40 ± 14.7 (*n* = 12)	33.75 ± 10.14 (*n* = 23)	N/A
Don't know	44.44 ± 10.02 (*n* = 12)	35.15 ± 10.70 (*n* = 21)	N/A

^*^ Statistically significant (*p* < 0.05) compared with allied health professionals and control group.

^#^ Statistically significant (*p* < 0.05) compared with control group.

To allow subgroup analyses between different domains of sleep knowledge, total sleep knowledge scores were divided between questions covering the domains sleep physiology and sleep disorders. All three groups demonstrated lower scores on questions about symptoms and characteristics of sleep disorders, compared with questions related to basic sleep physiology (Table 2). Subgroup analysis revealed different scores across groups on both domains, with RPs scoring significantly higher than AHPs (sleep physiology *p* < 0.001*; sleep disorders *p* < 0.001*) and controls (sleep physiology *p* = 0.002*; sleep disorders *p* < 0.001*). Controls showed higher scores than AHPs within the domain sleep physiology, but this difference failed to reach statistical significance after correcting for alpha (*p* = 0.034). Within the domain sleep disorders, AHPs demonstrated significantly higher scores than controls (*p* = 0.013*).

#### 3.4.2 Sleep hygiene

**FIGURE 2:**
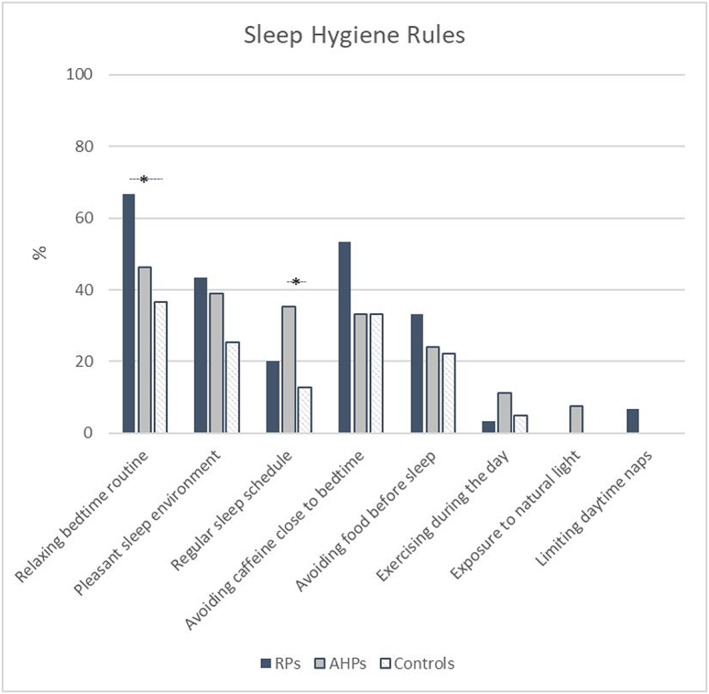
Frequency of sleep hygiene rules mentioned by RPs (*n* = 30), AHPs (*n* = 54) and controls (*n* = 63), in addition to the example rule regarding limiting screen time 2 h before bedtime. * *p* < 0.05 (Fisher's exact test); AHPs, allied health professionals; RPs, rehabilitation physicians [Colour figure can be viewed at wileyonlinelibrary.com]

The frequency of sleep hygiene rules mentioned by RPs, AHPs and controls is shown in Figure 2. RPs were able to more frequently recall the majority of sleep hygiene rules. Significant differences were observed between groups for *establishing a relaxing bedtime routine* (*p* = 0.015*, Fisher's exact test) and *establishing a regular sleep schedule* (*p* = 0.026*, Fisher's exact test), with these rules being mentioned more often by RPs and AHPs compared with controls, respectively. The sleep hygiene rules closest to bedtime and related to the sleep environment were best known across groups, whereas those related to daytime practices (i.e., *promoting physical exercise during the day*, *exposure to natural light* and *limiting daytime naps*) were rarely mentioned across all groups.

#### 3.4.3 Self‐perceived sleep knowledge

The minority of all HCPs (RPs 20%, *n* = 6; AHPs 14.8%, *n* = 8) reported to believe that they possess sufficient sleep knowledge to address sleep problems in daily clinical practice. In contrast, most HCPS reported either to not have sufficient knowledge (RPs 40%, *n* = 12; AHPs 42.6%, *n* = 23) or that they ‘*do not know*’ (RPs 40%, *n* = 12; AHPs 38.9%, *n* = 21) (Table 2).

No difference was found in total sleep knowledge scores between HCPs who believed they had sufficient knowledge about sleep versus those who believed they did not have sufficient sleep knowledge, RPs: *t*(16) = 0.652, *p* = 0.524; AHPs: *t*(29) = 0.196, *p* = 0.846.

## 4 DISCUSSION

This study assessed the sleep health practices and knowledge about sleep physiology, sleep disorders and sleep hygiene among two groups of HCPs in Dutch paediatric rehabilitation. The frequency of sleep being addressed during clinical encounters varies greatly between medical and non‐medical HCPs, and more efforts should be made for sleep assessments to become a standard item for surveillance during routine healthcare practices. Although RPs showed higher sleep knowledge scores than AHPs, neither group exceeded 50% correct scores, suggesting limited sleep knowledge, particularly in the area of sleep disorders. We also noticed limited familiarity with healthy sleep behaviours that can be practiced during the day. Our findings emphasize the need to educate and empower HCPs with sound knowledge, skills and confidence required to address sleep problems in children with NDDs and to support their parents.

There are several strengths and limitations to this study that should be considered. The questionnaire used was developed in co‐creation with researchers and clinicians in the field of paediatric rehabilitation and sleep medicine, based on currently available literature in these fields, and trialled before use, yet it should be noted that it has not been validated. HCPs may have felt obliged to respond favourably to questions concerning their sleep health practices. Also, the relatively small sample size may limit the generalisability of our findings. Unlike other survey studies on sleep knowledge levels, we did include a control group to allow comparisons of HCP scores to the general population.

In line with our results, survey studies of practicing physicians and paediatricians have consistently found poor knowledge about sleep and significant gaps in clinical practices regarding paediatric sleep disorders (BaHammam, 2000; Bruni et al., 2004; Gruber, Constantin, Frappier, Brouillette, & Wise, 2017; Owens, 2001; Papp, Penrod, & Strohl, 2002). For example, Bruni et al. found low scores in all areas of sleep knowledge and particularly in sleep disorders among paediatricians and child neuropsychiatrists (Bruni et al., 2004). Papp and colleagues reported an average knowledge of 34% among primary care physicians, with only 10% rating themselves as *good* (Papp et al., 2002). Similarly, we found the self‐perceived knowledge among paediatric rehabilitation professionals to be low, that is, only one in five RPs, and one in seven AHPs rated their own sleep knowledge to be sufficient. Without proper training and experience, HCPs may lack confidence or feel incompetent to address sleep problems properly, resulting in sleep problems left unaddressed and untreated (Honaker & Meltzer, 2016). As more HCPs acquire a greater awareness of sleep, more consistent processes for screening and assessment can be developed across paediatric rehabilitation settings.

Given that parental knowledge about children's sleep is typically poor (McDowall, Galland, Campbell, & Elder, 2017), they should be provided with appropriate information and advices to ensure that healthy sleep practices are implemented and maintained at home (Blackmer & Feinstein, 2016; Mindell & Owens, 2003). Nearly all HCPs reported to give such advices on a frequent basis, yet their knowledge deficits are indicated by equally low total sleep knowledge scores as our control sample, which consisted mostly of parents. A recent study on sleep problems and solution seeking for children with cerebral palsy and their parents reported that out of the 63 parents that asked for professional help with their child's sleep, only 21 reported that their request for help led to effective treatment or advice from their HCP (Petersen et al., 2020). In addition, we found HCPs' familiarity with sleep hygiene rules to be confined to those closest to bedtime and related to the sleep environment, whereas they appeared unfamiliar to daytime practices (like exposure to daylight, adequate exercise and limiting daytime naps). This knowledge gap is worrisome since sleep hygiene is considered the first line of treatment for sleep problems in children with NDDs (Jan et al., 2008).

A review of the lifestyle practices that contribute to good quality sleep can be valuable in providing a starting point to improve sleep and more broadly in adopting healthy and protective lifestyles. In fact, promoting the entire triad of healthy behaviours, which in addition to physical activity and nutrition, also includes sleep itself, has recently been described as ‘the formula for health and well‐being’ in vulnerable patient populations with neurodevelopmental (Verschuren, McPhee, Rosenbaum, & Gorter, 2016) and neuropsychiatric disorders (Briguglio et al., 2020). Clearly, and in alignment with the global medical trend towards preventive healthcare (Egger, Binns, Rossner, & Sagner, 2017), the need to protect, promote and maintain healthy sleep as part of a healthy lifestyle, especially in children with NDDs, is evident. But if we want doctors to preach a healthy lifestyle to their patients (and families), expect them to detect and prevent sleep problems early on and require them to effectively guide parents, obviously they need to be adequately equipped with proper training before they enter the clinic.

Unfortunately, there is only very limited coverage of sleep education in medical schools, which has previously been identified in 409 medical schools across 12 countries (Mindell et al., 2011), and appears to persist. Consistent with their findings from nearly a decade ago, we found that the majority of Dutch physicians received less than 5 h of sleep education, similar to non‐medical professionals and controls. This alarmingly low number may in turn explain their limited sleep knowledge and feelings of incompetence to address sleep in clinical practice. Indeed, limited exposure to sleep education can predict medical trainees' confidence and knowledge levels, thereby forecasting their future clinical practices regarding sleep health (R. E. Salas et al., 2013). This advocates the continued need for sleep medicine education to be fully incorporated into medical school curricula (R. M. E. Salas et al., 2018).

Taking into account the confines of an already packed medical curriculum, additional educational efforts like postgraduate training, clinical opportunities and other sleep education tools for current HCPs are warranted. It has been shown that sleep knowledge can be successfully increased through provision of sleep education, both when delivered face‐to‐face and through online webinars (Osborne & Blunden, 2018). Our results showed that the knowledge scores of HCPs who believed to possess sufficient sleep knowledge did not differ from those who believed they lacked sleep knowledge, demonstrating the importance to undertake such sleep training programs regardless of self‐perceived knowledge. The goal of educational sleep trainings should not be to turn HCPs into sleep experts but rather to provide them with the knowledge required to recognize symptoms of major paediatric sleep disorders by asking the right questions, to give the right general advice regarding good sleep hygiene and to enable them to know when to refer to a (sleep) specialist for further assessment or to initiate sleep treatment strategies (Luginbuehl & Kohler, 2009).

## AUTHOR CONTRIBUTION

R. Y. H. made substantial contributions to conception and design of the study and acquisition and analysis of data and interpretation of data. She wrote the manuscript and approved of the final version to be published. S. P. was involved in the design of the study and data acquisition and analysis and made significant contributions to the manuscript. J. M. V. was involved in the data analysis and interpretation of the data and contributed to revision of the manuscript. N. R. made great contributions to data entering, data analysis and interpretation and was involved in drafting the article. J. M. A. V. contributed to conceptualization and interpretation of data and revising the manuscript for important intellectual content. O. V. was involved in the concept and design of the study, data analysis interpretation of data and contributed to drafting and revising the article. All persons who meet authorship criteria are listed as authors, and all authors certify that they have participated sufficiently in the work to take public responsibility for the content, including participation in the concept, design, analysis, writing or revision of the manuscript. All authors reviewed the text of the manuscript and gave final approval of the version to be published.

## CONFLICT OF INTERESTS

The authors have no conflicts of interest to declare.

## Supporting information



Supporting Information S1
