# Supplementary material for: Sleep health practices and sleep knowledge among healthcare professionals in Dutch paediatric rehabilitation
Source: Child Care Health Dev. 2020 Aug 12;46(6):703–10. doi: 10.1111/cch.12799 (PMC7589250; doi:10.1111/cch.12799)
Supplement: Supplementary file 1 — Supporting Information S1 [file CCH-46-703-s001.DOCX]

**Supplementary material - Appendix 1.** Sleep survey questionnaire

***Sleep survey***

***General information***

1. **What is your profession?**

- Paediatric rehabilitation physician
- Doctor in specialist training to become paediatric rehabilitation physician
- Paediatrician
- Physician assistant
- Physical therapist
- Occupational therapist
- Developmental behavioural therapist
- Speech and language therapist
- Social worker
- Psychologist
- Other: ______________________________________________

1. **Sex:**

- Female
- Male

1. **Age range:**

- 20-30 years
- 31-40 years
- 41-50 years
- 51-60 years
- 61-70 years

1. **How many hours of sleep education did you receive throughout your entire school curriculum?**

- 0-5 hours
- 5-10 hours
- 10-15 hours
- 15-20 hours
- > 20 hours

1. **Do you find this an adequate amount?**

- Yes
- No
- *Don’t know*

***Sleep Health Practices***

1. **Do you believe you have sufficient sleep knowledge to address sleep issues in clinical practice?**

- Yes
- No
- *Don’t know*

1. **How often do you address sleep-related issues in clinical practice?**

- Never/seldom (less than 1x per month)
- Sometimes (1-3x per month)
- Often (1x per week or more often)

1. **If *sometimes* or *often*:
   What kind of sleep therapies/interventions do you apply?** *(multiple answers are allowed)*

- Sleep medication (e.g. melatonin)
- Behavioural intervention
- Light therapy
- Advice (about sleep hygiene / healthy sleep practices)
- Referral to sleep clinic
- Other: ______________________________________________

***Sleep Knowledge***

1. **How many hours of sleep are recommended for adults?**Between _______ & _______ hours
2. **How many hours of sleep are recommended for a 4-year old child?**

Between _______ & _______ hours

1. **The sleep cycle of an adult lasts about:**

- 30 minutes
- 1 hour
- 90 minutes
- 2 hours
- *Don’t know*

1. **The sleep cycle of a 1-year old lasts about:**

- 15 minutes
- 30 minutes
- 45 minutes
- 1 hour
- *Don’t know*

1. **Peter is 3 years old and according to his parents he wakes up 2x per night. This is considered:**

- Less frequently than usual
- Normal
- More often than usual
- *Don’t know*

1. **REM-sleep is a sleep stage characterised by the occurrence of rapid eye movements. What is another typical characteristic sign of REM-sleep?**
   _________________________________________________________________

_________________________________________________________________

1. **Do children have more or less REM-sleep than adults?**

- More
- Less
- *Don’t know*

1. **Most REM-sleep occurs during which part of the night?**

- Early
- Mid
- Late
- *Don’t know*

1. **Most dee sleep occurs during which part of the night?**

- Early
- Mid
- Late
- *Don’t know*

1. **How does daylight affect sleep?**

- Daylight can cause one to sleep lighter
- Daylight can cause one to sleep earlier
- Daylight can cause one to sleep later
- Daylight can cause one to sleep earlier or later
- *Don’t know*

1. **What statement about melatonin as medication is true?**

- Melatonin makes you sleep deeper
- Melatonin should be taken 2 hours before sleeping
- Melatonin should be taken 30 minutes before sleeping
- Melatonin can change your day/night rhythm
- *Don’t know*

1. **What is the ideal room temperature for sleeping?**

- 16-18°C
- 18-20°C
- 20-22°C
- *Don’t know*

1. **What are characteristics of sensory overstimulation in relation to sleeping?** *(multiple answers are allowed)*

- Want to sleep, but cannot sleep
- Can sleep, but don’t want to sleep
- Waking up during the night
- A busy day = worse night
- *Don’t know*

1. **What are physical causes of insomnia?** *(multiple answers are allowed)*

- Snoring
- Disturbed melatonin levels
- Obstipation
- Pain
- Asthma
- *Don’t know*

1. **What are the signs that a child has insufficient sleep?** *(multiple answers are allowed)*

- Having to wake up the child in the morning
- Behavioural/concentration problems
- Sleeping in on weekend days
- Sleeping beyond nap times
- *Don’t know*

1. **Which conditions or symptoms are risk factors for obstructive sleep apnoea?** *(multiple answers are allowed)*

- Down syndrome
- Orofacial cleft (cleft lip and cleft palate)
- Asthma
- Autism
- Generalized hypotonia
- *Don’t know*

1. **How can you distinguish nightmares from sleep terrors (night terrors)?**

- Sleep terrors are scarier than nightmares
- Sleep terrors typically occur during the first half of the night
- Children usually remember their sleep terrors better in the morning, compared to nightmares
- Sleepwalking only occurs in nightmares, not in sleep terrors
- *Don’t know*

1. **Sleepwalking occurs during:**

- REM-sleep
- non-REM-sleep stage 1
- non-REM-sleep stage 2
- non-REM-sleep stage 3
- *Don’t know*

1. **What symptom(s) is/are characteristic for children with obstructive sleep apnoea syndrome?**

- Snoring
- Busy behaviour
- Bedwetting
- All the above symptoms
- *Don’t know*

1. **An important sleep hygiene practice is to limit screen time (e.g. TV/phone)
   two hours before bedtime. Name three other sleep hygiene rules:**

1. _______________________________________________________________

2. _______________________________________________________________

3. _______________________________________________________________
